# Development and Status of the National Oral Health Surveillance System

**Published:** 2009-03-15

**Authors:** Laurie Barker, Dolores M. Malvitz, Kathy R. Phipps

**Affiliations:** Surveillance, Investigations and Research Team, Division of Oral Health, National Center for Chronic Disease Prevention and Health Promotion, Centers for Disease Control and Prevention; Decatur, Georgia; Association of State and Territorial Dental Directors, Morro Bay, California

## Abstract

During the last 2 decades of the 20th century, few national, state, or local oral health programs were able to conduct public health surveillance in a timely fashion. Under the leadership of the Association of State and Territorial Dental Directors and with substantial support from the Division of Oral Health at the Centers for Disease Control and Prevention, the National Oral Health Surveillance System was established as a first step in helping oral health programs routinely document population needs and program impact with standard, feasible methods. In 1999, the Council of State and Territorial Epidemiologists approved 7 oral health indicators for public health surveillance: 3 for adults (most recent dental visit, most recent dental cleaning, total tooth loss) using data from the Behavioral Risk Factor Surveillance System; 3 for third-grade students (presence of treated or untreated dental caries, untreated tooth decay, dental sealants) collected by states using a standard screening protocol; and the percentage of the population served by public water systems that receives optimally fluoridated water, tracked through the Water Fluoridation Reporting System. The Web site that describes the National Oral Health Surveillance System (http://www.cdc.gov/nohss/) and provides access to current indicators was launched in 2001 with adult and water fluoridation data for all states; child indicators were added later. Data are now available electronically for 35 to 51 states (including the District of Columbia), depending on the indicator, indicating progress toward state-specific monitoring of these oral health indicators.

## Introduction

Until recently, dental programs have focused little attention on public health surveillance, which has been defined as “the ongoing systematic collection, analysis, and interpretation of outcome-specific data for use in the planning, implementation, and evaluation of public health practice” (1). Although surveillance data may identify research and service needs, public health surveillance is not, itself, epidemiologic research (2,3). Thus, effective surveillance requires 1) the capacity for data collection, analysis, and interpretation; 2) timely dissemination of the information derived from data to people who can undertake effective prevention and control activities; 3) a focus on tracking specific health outcomes, rather than only intermediate behaviors or process measures of program activity; and 4) decision making for programs and policies based on current data, especially on trends over time (1).

Since health objectives for the United States first were created in 1979, oral health objectives have been included as a separate focus area (4). However, during the last 2 decades of the 20th century, few state or local programs to improve oral health had the capacity to monitor progress toward those objectives. State-specific data to characterize children's oral health status or behaviors were uncommon, and corresponding data for adults did not exist. In dentistry, no records systems are widely used that are comparable to vital records or diagnosis codes taken from insurance claims and hospital discharge data. Although oral health has been monitored at the national level using health surveys of the National Center for Health Statistics, Centers for Disease Control and Prevention (CDC), or oral health surveys conducted episodically during the 1970s and 1980s by the National Institute of Dental and Craniofacial Research, all but a few state oral health programs lacked both a surveillance system and the capacity to conduct public health surveillance.

This absence of state or local data stemmed primarily from the methods that had evolved for monitoring diseases of the oral cavity; national examination surveys of oral health used complex sampling protocols, clinical evaluation by a dentist, and multiple detailed measures for each tooth. Analysis also was complex, often stretching the interval between primary data collection and dissemination to several years. Although these "gold standard" methods had been developed for epidemiologic research, the dental community expected to use them for program planning and evaluation as well. Given the methods' complexity and expense, however, few oral health programs — even at the national level — accomplished public health surveillance in a timely fashion. Thus, in practice, the prevalence of oral disease among at-risk populations at the state level remained largely undocumented.

## National Oral Health Surveillance System

In response to that dearth of state data, the National Oral Health Surveillance System (NOHSS) was established at the turn of the 21st century as a first step in helping oral health programs in state health agencies meet expectations that they routinely document population needs and program impact, for example, to track progress toward *Healthy People 2010* objectives, for allocation of funding from the Maternal and Child Health and Preventive Health and Health Services block grants, to justify allocation of discretionary resources, or to evaluate programs or policies. In 1999, at the annual meeting of the Council of State and Territorial Epidemiologists (CSTE), its members approved 7 oral health indicators for surveillance (Table). That same year, CSTE also released standard definitions for 73 chronic disease indicators (5) that had been approved conceptually 2 years earlier, including several of particular interest to the dental community, for example, incidence of and mortality from cancer of the oral cavity or pharynx, prevalence of cigarette smoking among adults and youth, use of smokeless tobacco among youth, and adult diabetes prevalence.

Critical leadership in establishing oral health surveillance came from the Association of State and Territorial Dental Directors (ASTDD), particularly its officers and members of its data committee. In 1992, ASTDD began efforts to standardize oral health questions that a few states had added to their surveys for the Behavioral Risk Factor Surveillance System (BRFSS). By 1994, BRFSS coordinators had approved a 4-question optional module, some or all of which 48 states used from 1995 through 1998; on the basis of those experiences, the module was revised to 3 questions and then approved for inclusion on the 1999 BRFSS emerging core questionnaire.

Much of the work that served as a foundation for NOHSS occurred under a cooperative agreement between ASTDD and CDC's Division of Oral Health (DOH) that began in 1997. One initiative, led by the Ohio state dental director with advice from a group of 30 content experts, focused on identifying simple methods to collect prevalence data and on training screeners (not necessarily dentists) to use standard protocols, case definitions, and criteria. Following evaluation of the reliability and validity of this Basic Screening Survey, the project culminated in ASTDD's 1999 publication of a manual, with data entry and analysis programs as well as a training video (6).

A second work group began meeting in September 1998; its charge was to shape the purpose and operation of what became the NOHSS. Some group members brought experience with surveillance methods used in the broad public health community, for example, 2 state-based chronic disease epidemiologists; others were opinion leaders within the specialty of dental public health. Multiple influences and events provided the impetus for action and reasonable models to follow (2,5,7-12). Work group members made rapid progress over the ensuing 9 months: from a list of 72 measures, they chose a "minimal list" that was further narrowed to the 7 indicators (all related to *Healthy People 2010* objectives) for which they sought CSTE approval (Table). The group identified 3 data sources: 1) The BRFSS for the 3 adult indicators; 2) prevalence data for third-grade students collected within the states, consistent with ASTDD's Basic Screening Survey, for all 3 child indicators; and 3) the Water Fluoridation Reporting System for fluoride status of community water supplies.

DOH contributed to these surveillance development efforts by 1) determining the validity of assessment innovations in states (13) and applying them in new settings (14,15), 2) including state-based surveillance as 1 of the major tasks of the 1997 cooperative agreement with ASTDD, 3) supporting implementation of state-based surveillance using the oral health indicators and funding research on alternative methods (16-20), and 4) educating the oral health community about surveillance approaches in other state health agency programs (3).

In 2000, shortly after CSTE approved the oral health indicators for public health surveillance (and thus, the concept of a national surveillance system for oral health), work began to create a Web site; CDC and ASTDD scientists organized existing data on the indicators and created templates for their visual presentation. The NOHSS work group, through consensus and e-mail, made major decisions. When it concluded that the Web site should be hosted at CDC, DOH scientists obtained appropriate approvals and funding to support the site. The first phase of the Web site presented indicators of adult oral health and water fluoridation, and data for all states were launched January 23, 2001. Over subsequent years, data for children's oral health indicators were added; to ensure comparability, the NOHSS work group had established a review process and explicit criteria for data submission, executed by ASTDD's lead epidemiology consultant. CDC has evaluated and revised the site (21) twice, and additional data for these indicators have been added when available. For all changes, both the ASTDD data committee and the NOHSS work group provided advice, review, guidance, and feedback.

Given that nearly 10 years have elapsed since CSTE approved the 7 oral health indicators, it seems appropriate to examine progress toward universal use of these measures by states and in published surveillance reports. Such information can be the basis for considering revision of indicators (21).

## Adult Oral Health Indicators

After obtaining data on adults for all states during 1999, DOH continued to fund an Oral Health Optional Module for BRFSS, even though few states used its questions in 2000 and 2001. BRFSS coordinators approved repeating the 3 oral health questions on the 2002 rotating core questionnaire and, since then, including them on that section of the BRFSS survey in 3 additional years (2004, 2006, 2008). Thus, data are available for the 3 adult indicators for all states for these 5 years, and 48 states have data for at least 1 earlier year from 1995 through 1998, for 2 of the indicators (past-year dental visit, complete tooth loss). The most recent revision of the chronic disease indicators (22) includes the 3 oral health indicators in the section Other Diseases and Risk Factors.

Prompt reporting of BRFSS findings has remained a DOH priority — a *Morbidity and Mortality Weekly Report *(*MMWR*) article on self-reported tooth loss for 48 states was published in 1999 as the lead for the World Health Day issue on aging (23), with substantial press coverage. Oral health data were included in 2 *MMWR* *Surveillance Summaries* (24,25) that focused on an array of indicators and behaviors among selected populations. *MMWR* also published state-specific data on retention of teeth and total tooth loss (edentulism) (26) and on dental visits among dentate adults with diabetes (27). Although analyses of BRFSS oral health data often are presented at professional meetings, publication in peer-reviewed journals remains less common. BRFSS data were used to examine California's progress toward the *Healthy People 2010* objective on adult dental visits (28), the association of tooth loss with heart disease (29), and the association of diabetes and tooth loss (30).

## Water Fluoridation Indicator

When the water fluoridation indicator was approved in the summer of 1999, efforts were under way within DOH to develop a Web-based, real-time system for monitoring water fluoride status and quality as a replacement for mailed survey methods used to produce past episodic reports. The new system was built on an existing database maintained by the Drinking Water Program of the Environmental Protection Agency (EPA), which regulates the safety and quality of water systems. Although this wide collaboration (among EPA, CDC, and state health agency stakeholders) presented challenges, the Water Fluoridation Reporting System (WFRS) became operational in January 2000. After verification of data quality, the first *MMWR* report of state-specific data on water fluoridation that used WFRS was published in early 2002 (31).

Because WFRS started with an existing EPA monitoring system, unique methodologic issues have arisen. States update their own information in WFRS, directly and regularly (at different intervals — as frequently as daily and as seldom as yearly), and DOH completes ongoing and annual assessments to enhance the quality of data in the system. Although the WFRS database contains information for all states and the District of Columbia, only 36 states have allowed access to their water fluoridation information on the public Web site (32). WFRS data are used both to identify recipients of annual awards for fluoridation operational excellence and to determine states' achievement of the *Healthy People 2010* water fluoridation objective. Recent methodologic work has focused on how different protocols affect the calculation of what percentage of a state's population that receives public water  supplies receives fluoridated water (33). DOH expects to publish reports from WFRS data every 2 years.

## Child Oral Health Indicators

The NOHSS Web site (21) displays data from 35 states for the 3 child indicators. These data were collected at different times — the oldest (2 states) date from the 1998-1999 school year and the most recent from 2005-2006 (1 state). Median school year of collection for currently posted data is 2002-2003, and reported response rates range from 32% to 99% (median, 64%). Two doctoral-level epidemiologists, with funding from the ASTDD cooperative agreement, have provided technical assistance on Basic Screening Survey methods to 25 states during the past 7 years. The remaining 10 states gathered data for the child indicators without such help (34,35).

At the time these child indicators gained approval, CSTE members expressed concern regarding the ability of states — many with limited capacity in their oral health programs — to collect, analyze, and disseminate data. Although the Basic Screening Survey streamlined complex methods for monitoring oral health, current child indicators still rely on collection of primary data rather than on use of ongoing systems. Neither ASTDD nor DOH has recommended an ideal interval for collection of child data, and states have not synchronized their efforts to conduct data collection in the same years. As long as primary data collection is required, however, it seems unlikely that any state will obtain state-specific data more frequently than every 5 years or that the NOHSS Web site will display data for a given year from more than 6 to 9 states.

## Use of the Oral Health Indicators

In 2000, the Surgeon General noted the intertwined nature of oral health and general health, the magnitude of disparities in oral health status among population groups within the United States, and the existence of effective disease prevention measures for most oral diseases (36); that report and a later report, *National Call to Action to Promote Oral Health* (37), included recommendations for improving the public health infrastructure, such as obtaining data for monitoring the oral health of populations. Recent national data (38) still reveal large disparities in oral health status between people from families with incomes of less than the federal guidelines for poverty (poor) and their peers from families with incomes of at least 200% of the federal poverty guidelines (nonpoor). For people of all ages, the prevalence of untreated tooth decay among people from poor families is twice that found among the nonpoor; the prevalence of dental sealants (a preventive intervention) among school-aged children from poor families is only half that of their nonpoor peers (38).

Anecdotal reports suggest that state-specific data have proved important in supporting continuation or expansion of state programs to address such disparities and improve oral health. At a March 27, 2007, hearing of the US House of Representatives Subcommittee on Health, called in response to the death of a Medicaid-eligible child because of complications arising from a severe, but preventable, case of dental disease, 3 state representatives presented estimates for the NOHSS child oral health indicators from their states; these estimates were results from state oral health surveys conducted using the Basic Screening Survey protocol. It is unlikely that these state data would have been available in the absence of NOHSS. State policies and programs, however, vary both in scope and priorities — which, in turn, frequently depend on funding sources. Use of the oral health indicators can help states monitor their progress toward *Healthy People 2010* objectives and determine the effectiveness and efficiency of different interventions. The 33 states that provide data on the NOHSS Web site for water fluoridation and child indicators (not the same 33 for each) have taken a first step. Lack of participation may stem from myriad reasons, for example, difficulty gaining approval at the state level to implement WFRS fully, lack of adoption of the child indicators among states with longstanding and unique methods for monitoring oral health, or inadequate oral health program and epidemiology capacity within the state health agencies.

Use of these data for additional state-based analyses and for decisions on program changes, however, requires both strong leadership from the state dental director and epidemiology capacity dedicated to the oral health program. Although the 12 states that receive DOH funding for infrastructure development are required to have at least a 0.25 full-time equivalent epidemiologist dedicated to oral health, such expertise remains uncommon among other oral health programs within state or local health agencies.

Although the past decade's success in establishing oral health surveillance has been noteworthy, ASTDD and DOH should continue applied research and evaluation, both within the states and at the national level, with particular attention to the utility and validity of methods that do not require primary data collection (39). ASTDD and DOH periodically should review the oral health indicators in light of changes in data availability, state experiences, and public health priorities.

## Figures and Tables

**Table.** Selected Characteristics of Oral Health Indicators for Surveillance Approved by the Council of State and Territorial Epidemiologists, 1999

Indicator	Complete Description	Data Source	No. of States^a^ With Data in NOHSS	Chronic Disease Indicator	*Healthy People 2010* Objective for Which State Data Could Be Used
**Adults**					
Dental visit	Percentage of adults aged ≥18 y who reported visiting a dentist or dental clinic in the past year	BRFSS	51	90	21-10^b^
Teeth cleaning	Percentage of dentate adults aged ≥18 y who reported having their teeth cleaned in the past year	BRFSS	51	91	Related to 21-5
Complete tooth loss	Percentage of adults aged ≥65 y who have lost all their natural teeth due to tooth decay or gum disease	BRFSS	51	92	21-4^c^
**Children**					
Dental caries experience	Percentage of third-grade students with any caries experience (ie, both treated and untreated tooth decay)	BSS^d^ statewide oral health screenings	33	NI^e^	21-1^b^
Untreated tooth decay	Percentage of third-grade students with obvious tooth decay that has not been treated	BSS statewide oral health screenings	33	NI^e^	21-2^b^
Dental sealants	Percentage of third-grade students with dental sealant present on at least 1 permanent molar tooth	BSS statewide oral health screenings	33	NI^e^	21-8^a^
**Community**					
Water fluoridation status	Percentage of people served by public water systems who receive optimally fluoridated water	WFRS	51/33^f^	NI^e^	21-9^g^

Abbreviations: NOHSS, National Oral Health Surveillance System; BRFSS, Behavioral Risk Factor Surveillance System; BSS, Basic Screening Survey; NI, not included; WFRS, Water Fluoridation Reporting System.

a The 50 US states and the District of Columbia.

b Data are available from BRFSS for selected local areas.

c Data from the same BRFSS question also can track *Healthy People 2010* objective 21-3, adults aged 35-44 y who have had no teeth removed due to disease.

d Statewide oral health screenings conducted using protocols consistent with the BSS (6).

e Indicator is not included in the chronic disease indicators project (5).

f Statewide data for 51 states are available in the WFRS database, and NOHSS and Oral Health Maps public Web sites. However, 33 states provide access to their most current WFRS data for counties and water systems through the public Web sites My Water's Fluoride (http://apps.nccd.cdc.gov/MWF/Index.asp) and Oral Health Maps (http://apps.nccd.cdc.gov/gisdoh/default.aspx).

g States may be able to use WFRS data to track *Healthy People 2010* objective 21-9 at local levels.
